# The Role of *Brachypodium distachyon* Wall-Associated Kinases (WAKs) in Cell Expansion and Stress Responses

**DOI:** 10.3390/cells9112478

**Published:** 2020-11-14

**Authors:** Xingwen Wu, Antony Bacic, Kim L. Johnson, John Humphries

**Affiliations:** 1 School of BioSciences, University of Melbourne, Parkville 3010, Victoria, Australia; 2 La Trobe Institute for Agriculture and Food, La Trobe University, Bundoora 3086, Victoria, Australia; t.bacic@latrobe.edu.au (A.B.); k.johnson@latrobe.edu.au (K.L.J.); 3 Sino-Australia Plant Cell Wall Research Centre, School of Forestry and Biotechnology, Zhejiang A&F University, Hangzhou 311300, China

**Keywords:** abiotic stress, biotic stress, cell expansion, plant defense, signaling, wall-associated kinase

## Abstract

The plant cell wall plays a critical role in signaling responses to environmental and developmental cues, acting as both the sensing interface and regulator of plant cell integrity. Wall-associated kinases (WAKs) are plant receptor-like kinases located at the wall—plasma membrane—cytoplasmic interface and implicated in cell wall integrity sensing. WAKs in *Arabidopsis thaliana* have been shown to bind pectins in different forms under various conditions, such as oligogalacturonides (OG)s in stress response, and native pectin during cell expansion. The mechanism(s) WAKs use for sensing in grasses, which contain relatively low amounts of pectin, remains unclear. *WAK* genes from the model monocot plant, *Brachypodium distachyon* were identified. Expression profiling during early seedling development and in response to sodium salicylate and salt treatment was undertaken to identify WAKs involved in cell expansion and response to external stimuli. The *BdWAK2* gene displayed increased expression during cell expansion and stress response, in addition to playing a potential role in the hypersensitive response. In vitro binding assays with various forms of commercial polysaccharides (pectins, xylans, and mixed-linkage glucans) and wall-extracted fractions (pectic/hemicellulosic/cellulosic) from both *Arabidopsis* and *Brachypodium* leaf tissues provided new insights into the binding properties of BdWAK2 and other candidate BdWAKs in grasses. The BdWAKs displayed a specificity for the acidic pectins with similar binding characteristics to the AtWAKs.

## 1. Introduction

As sessile organisms, plants have sophisticated strategies to sense and respond to both environmental and developmental signals in order to adapt their growth. Environmental stresses include abiotic and biotic stresses that cause loss of cell wall integrity or damage. The perception of cell wall breakdown is essential for plants to survive these stresses [1,2,3]. In addition to wall damage, plants must maintain integrity during normal growth and development in order to generate the turgor required for anisotropic growth as well as retain structural integrity. The cell wall therefore plays a critical role in signaling responses, acting as both the sensing interface and regulator of plant cell integrity [4,5,6,7].

Cell wall sensors that interpret stress experienced in the cell wall and/or at the plasma membrane and transduce signals intracellularly are proposed to be integral to responses to maintain wall integrity [8,9,10]. Within the receptor-like kinase (RLK) family, various subfamilies with a range of putative ligand-binding extracellular regions have been identified with roles in plant cell wall signaling [11,12]. For example, perception of cell wall defects associated with impaired cellulose synthesis is regulated by FEI1 and FEI2 from the leucine-rich repeat receptor kinase (LRR-RLK) family [13,14], along with THESEUS1 from the *Catharanthus roseus* protein kinase 1 like receptor kinase (CrRLK1L) family [15]. Wall-associated kinases (WAKs) are also cell wall-related signaling RLKs implicated in cell wall integrity sensing. WAK members in *Arabidopsis thaliana* have been shown to interact with cell wall pectins and participate in cell expansion and stress responses [16,17,18]

In *A. thaliana*, five tandemly arrayed *WAK* genes have been identified [16,19]. Members of this RLK subfamily typically contain a Ser/Thr kinase domain, and an extracellular domain with two epidermal growth factor (EGF)-like repeats [16,20]. A further 21 *WAK-like* genes (*WAKL*) are present in *A. thaliana*. *WAKL* genes are similar to *WAK*s, but encode proteins which are truncated or possess variations to the arrangement of extracellular domain features of WAKs. *AtWAK* genes have distinct, but overlapping expression profiles, with some exhibiting the highest expression levels in expanding tissues [17], suggesting a role for these WAKs in regulating cell expansion. Various environmental stimuli are able to induce the expression of *AtWAKs*—such as sodium salicylate (NaSA) treatment [21], physical wounding [17,22], and mineral toxicity [23]—indicative of WAKs involvement in plant defensive responses. While the precise mechanism of WAKs mediation of the signal for plant growth and immunity remains unclear, it is proposed that this signaling pathway in dicots is triggered through a covalent interaction between WAKs and pectins [17,18,24,25]. 

Several studies of grass species have implicated WAKs in the immune responses of wheat [26], maize [27], and rice [28,29], as well as in cell expansion and development [30,31]. In particular, non-RD WAKs, those devoid of a conserved Arginine(R)/Aspartate(D) motif in the kinase domain, have been shown to be involved in pathogen response [32]. However, there is still much to be learned regarding the mechanism(s) WAKs use for sensing in grasses, specifically regarding their interactions with pectins since the wall pectin content of the commelinid monocots (that include the grasses) is much lower compared to that observed in dicots [33,34,35,36]. In grass cell walls, the major non-cellulosic polysaccharides are heteroxylans and mixed-linkage β-glucans (MLGs). The question remains as to whether WAKs of grass species bind to either pectins or other polysaccharides. WAKs in *Arabidopsis* have been shown to bind pectins in different forms under various conditions, such as oligogalacturonides (OGs) in stress response, and native pectin during cell expansion. Although previous studies proposed roles for grass *WAK*s in both cell expansion and stress responses [37] detailed analysis of polysaccharide binding capabilities of grass WAKs has not been performed. 

In this study, *WAK* genes from the monocot plant, *Brachypodium distachyon* were investigated. Expression profiling during early seedling development and in response to (NaSA) and salt treatment was undertaken to identify WAKs involved in cell expansion and response to external stimuli. A number of candidate *BdWAK* genes were investigated for roles during expansion and defence responses, with one gene (*BdWAK2*) displaying increased expression during both cell expansion and stress response, in addition to playing a potential role in the hypersensitive response. In vitro binding assays provide insight into the binding of BdWAK2 and other candidate BdWAKs with various forms of pectin, providing new information on the binding activities of WAKs in grasses.

## 2. Materials and Methods

### 2.1. Plant Materials

Seeds of *B. distachyon* (diploid inbred line Bd21) and *Nicotiana benthamiana* were planted in pots (3 plants/0.5 L pot for *B. distachyon*, 1 plant/0.35 L pot for *N. benthamiana*) filled with soil mix (80% soil, 20% perlite, 100 g Osmocote fertilizer per 20 L soil mix) and grown in a growth chamber (constant 20 °C, 16 h light/8 h dark cycles). For stress-induction experiments, the *B. distachyon* seedlings were grown hydroponically using a modified Hoagland Solution [38]. To initiate the stress responses, either NaSA or NaCl solutions were added to the solution for a final concentration of 0.5 mM NaSA and 250 mM NaCl. Treatment lasted for 72 h during which the nutrient solution and additive (NaSA or NaCl) was replaced every 24 h.

### 2.2. RNAseq Analysis of B. distachyon Coleoptiles

Coleoptiles of *B. distachyon* were excised at 48 h post-germination in batches of 30 coleoptiles per replicate (10 mg fresh weight) and RNA extracted using the ISOLATE plant RNA kit (Bioline, Australia). RNA quantity and quality were assessed by the Agilent 2200 Tapestation system. Three replicate RNA samples (>2 µg total RNA for each replicate) were processed by Novogene (China) for RNAseq analysis. The NEBNext^®^ Ultra™ II RNA Library Prep Kit for Illumina^®^ (New England Biolab Inc., Ipswich, MA, USA) was employed to convert RNA into high quality non-directional libraries for next-generation sequencing on the Illumina^®^ platform. The original raw data from Illumina HiSeq 2500 platform was transformed to sequenced reads by base calling, generating 150 bp paired end reads. Clean reads, after quality control, were de novo assembled for transcriptome reconstruction using the bioinformatic platform Trinity [39].

### 2.3. Protein Sequence and Phylogenetic Analysis

Nucleotide and protein sequence analysis were performed using NCBI BLAST (http://blast.ncbi.nlm.nih.gov) [40] and Pfam (http://pfam.xfam.org) [41]. Genes were annotated using iTAK (http://bioinfo.bti.cornell.edu/cgi-bin/itak/index.cgi) and Ensembl Plants (http://plants.ensembl.org). Nucleotide or protein alignment were performed with Geneious (version 5.6.6) [42], using global alignment with free end and gaps, with gap open penalty at 12; gap extension penalty at 3; refinement iteration at 2. For phylogenetic analysis, the neighbor-joining method [43] was used, and the genetic distance was calculated using Jukes–Cantor model [44].

### 2.4. Quantitative PCR and Data Analysis

RNA was extracted using ISOLATE Plant RNA Kit (Bioline, Eveleigh, Australia) following the manufacturer’s instructions. One ug of RNA was used in each cDNA synthesis reaction utilising SuperScript III reverse transcriptase (Life Technologies, Carlsbad, CA, USA). Quantitative PCR (qPCR) was performed using method described by [45] with minor modifications. Samples were initially denatured at 95 °C for 10 min followed by 45 cycles according to the following profile: 95 °C for 10 s, 58 °C for 30 s, 72 °C for 20 s, 80 °C for 20 s. Reference genes in *B. distachyon* (*Glyceraldehyde-3-phosphate dehydrogenase* [*BdGAP]*, *Elongation factor-1α* [*BdELF*], *BdTubulin*), and *N. benthamiana* (*NbActin*, *NbUbiquitin*) were utilised for this study. For stress-induction experiments, control genes were also used as listed: NaSA-responsive gene (*Required for MLA12 resistance 1, BdRAR1*); NaCl-responsive gene (*Responsive to dehydration 22*, *BdRD22*); programmed cell death (PCD) marker gene (*Cell death marker, NbCDM*). Primers used in this study are displayed in Table S1. qPCR reactions were performed using SensiMix SYBR No-ROX (Bioline, Australia) according to the manufacturer’s instructions. Experiments were performed in triplicate with three biological replicates of each sample cDNA using a Rotor-Gene RG3000 (Corbett Research, Sydney, Australia) thermocycler. A geNorm method described by [46] was adopted to normalize the data to reference genes.

### 2.5. PCR Amplification and Cloning of Genes of Interest

Primers (Table S1) were designed to amplify the desired portion of the open reading frame (ORF) of selected *WAK* genes, as well as the *A. thaliana* BRI1 kinase domain, which was amplified from a full-length AtBRI1:GFP construct kindly provided by Professor Joanne Chory (SALK Institute for Biological studies, USA) [47]. KOD Hot Start DNA Polymerase (EMD Millipore, North Ryde, Australia) was used for the amplification, according to the manufacturer’s instruction. PCR-amplified WAK extracellular domains and kinase domains (in addition to the AtBRI1 kinase domain) were cloned into the Gateway entry vector pCR8GWTOPO (Invitrogen, Carlsbad, CA, USA), and subsequently transferred into pUB::CGFP [48] for protein expression in *N. benthamiana* and/or pDEST17 (Life Technologies, Eugene, OR, USA) for protein expression in *E. coli* where appropriate through Gateway technology [49]. 

### 2.6. Expression of Recombinant Proteins in E. coli and Protein Purification

Constructs generated for recombinant protein expression were transformed into *E. coli* BL21 (DE3) (Life Technologies, USA). Protein expression was induced in cultures with OD600 of 0.6 (mid-log phase) using 0.4 mM isopropyl β-D-1-thiogalactopyranoside (IPTG) at 28 °C for 4 h. Cells were collected by centrifugation and resuspended in 3 mL ice cold lysis buffer (50 mM Tris-HCl pH 8.0, 0.2 M NaCl, 10% (*v*/*v*) glycerol, 1% (*v*/*v*) Triton-X-100, 1× proteinase inhibitor cocktail (Roche, Mannheim, Germany). Resuspended cells were lysed twice on ice with 2 min sonication using a Bandelin SONOREX (Sigma-Aldrich, Sydney, Australia) sonication bath. Following cell lysis, cell pellets containing inclusion bodies were collected and 5 mL of inclusion body solubilisation buffer (50 mM Tris-HCl pH8.0, 0.2 M NaCl, 6 M guanidine-HCl, 1 mM DTT, 1× proteinase inhibitor cocktail (Roche, Germany) were added to the pellet and mixed at room temperature until the pellets solubilised. Affinity chromatography was used to enrich the His-tagged recombinant proteins using cOmplete Ni-NTA purification resin (Roche, Germany) in a 20 mL chromatography column according to manufacturer’s instructions. 

### 2.7. Kinase Activity Assay with Pro-Q Staining

The kinase domains fused to poly-His were expressed and enriched as described above in Section 2.6. Protein samples were separated via SDS-PAGE. Following fixation (50% (*v*/*v*) methanol, 10% (*v*/*v*) acetic acid), Pro-Q Diamond phosphoprotein gel staining solution (Thermo Fisher Scientific, Waltham, MA, USA) was used to stain the gel in darkness for 2 h according to manufacturer’s instructions, and the phosphoprotein signal was observed under UV light using ChemiDoc MP Imaging System (Bio-Rad, Gladesville, Australia). The same gel was subsequently stained with Coomassie Blue Silver staining solution (0.12% (*w*/*v*) Coomassie G-250, 10% (*w*/*v*) ammonium sulfate, 10% (*v*/*v*) phosphoric acid, 20% (*v*/*v*) methanol) for 2 h, and the gel imaged using a Bio-Rad Gel-Doc system. A method previously described [50] was adopted to analyse and quantify the kinase activity using Image Lab software version 5.2.1 (Bio-Rad, Australia). 

### 2.8. Cell Wall Polysaccharide Extraction and In Vitro Binding Assay

Pectins, hemicellulose, and cellulose were extracted from a preparation of alcohol insoluble residues (AIR) as previously described [51]. Polysaccharides tested include pectin (citrus, Sigma, St Louis, USA), type I arabino-4-galactan (larch wood, Sigma, St Louis, USA), low and high methyl-esterified pectin (citrus, Megazyme, Sydney, Australia), xylan (birch wood, Poly(β-D-xylopyranose), Sigma, USA) and mixed-linkage glucan (extracted from barley, using method described in [52]). Oligogalacturonides (DP > 9) was prepared from 1% polygalacturonic acid (85% de-esterified, Sigma, USA) following the protocol previously described [53]. WAK-polysaccharide interactions were analysed using a modified enzyme-linked immunosorbent assay (ELISA) as described previously [18]. Microplate wells were initially pre-treated with 50 μg/mL poly-L-lysine Hydrobromide (Sigma, USA) for 60 min. Polysaccharide (50 μL of 200 μg/mL dissolved in 0.5 mM Ca^2+^/150 mM Na^+^ Tris buffer) was subsequently applied to each well and incubated overnight at 4 °C to coat the wells. Wells were blocked with 3% milk powder dissolved in 0.5 mM Ca^2+^/150 mM Na^+^ Tris buffer (room temperature for 2 h). Recombinant WAK domain protein (250 ng) was then added to the wells (room temperature for 2 h), which were subsequently washed thoroughly to remove unbound protein. Subsequently a 6x-His Epitope Tag Antibody (Thermo Fisher Scientific, USA) as primary antibody (1:1000 dilution) and goat anti-mouse horseradish peroxidase (HRP) as secondary antibody (1:10,000 dilution) were applied to detect His-tagged recombinant proteins remaining bound to the polysaccharides. Colorimetric 3,3′,5,5′-Tetramethylbenzidine (TMB) reagent (HRP substrate, Merck, New Jersey, USA) was used to measure absorbance at 450 nm with a Spectrostar Nano plate reader (BMG LABTECH, Mornington, Australia) to quantify the recombinant protein bound to the polysaccharide. Binding assays were performed in triplicate, and binding of each recombinant protein to wells with no polysaccharide added was analysed as the baseline measurement.

## 3. Results

### 3.1. Identification and Analysis of the BdWAK Family

**Figure 1:**
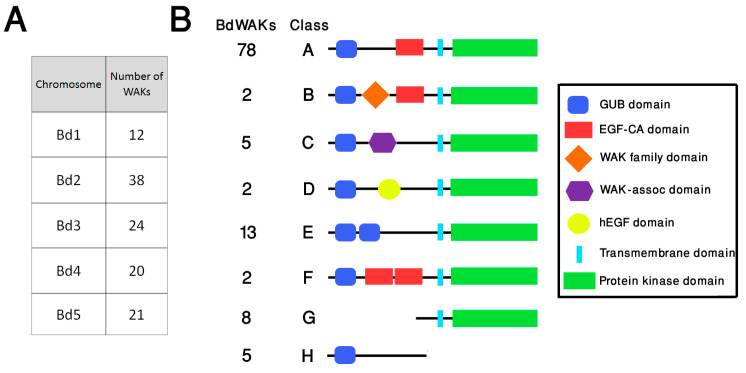
Organisation and structure of WAK members in *B. distachyon* (**A**) Distribution of *BdWAK* genes on *B. distachyon* chromosomes. (**B**) Schematic representation of eight structural classes (A–H) of WAK proteins identified in *B. distachyon*. The number of BdWAKs identified for each class is indicated. The eight classes have varying motifs and motif organisation with Class A considered the typical WAK structure and Class B through Class H considered ‘non-typical’ WAK structures. Class A: typical WAK structure with a GUB (galacturonan-binding) domain, an EGF-Ca (epidermal growth factor-like calcium-binding) domain, and a kinase domain; Class B: as for Class A, but with a WAK family domain present between the GUB and EGF-Ca domains; Class C: a WAK-associated domain is present after the GUB domain and there is no EGF-Ca domain; Class D: a variant on the Class C structure with a hEGF domain present instead of the EGF-Ca domain; Class E: contains a duplication of the GUB domain and lacks the EGF-Ca domain; Class F: contains a duplication of the EGF-Ca domain between the GUB and protein kinase domains.; Class G is a truncated protein with only a protein kinase domain; Class H is a truncated protein with only the extracellular GUB domain.

Bioinformatics studies of predicted WAK members in grasses have indicated that the number of members is larger than that found in dicots, such as *A. thaliana*. In agreement with this, database searching of the *B. distachyon* genome identified a total of 115 members (compared to 26 in *A. thaliana*) of the WAK family using published WAK/WAKLs as query sequences (Figure S1). Although WAKL sequences were included as query sequences in searching the *B. distachyon* genome, for clarity in this study all *B. distachyon* sequences which fit the broader definition of WAK/WAKL are referred to as BdWAKs. The 115 *BdWAK*s are distributed among all chromosomes, with chromosome 2 displaying the highest occurrence (38) (Figure 1A, Data file S1). Two clusters of *BdWAK* genes are apparent on chromosome 2 (Bd2:1635336-1700774 and Bd2:47168187-47198608), and a subset of these *BdWAK* sequences in close physical proximity display high sequence similarity to each other (green boxes in Figure S1).

Protein motif annotations show that these 115 members can be classified into eight structural classes (A to H) (Figure 1). In addition to the characteristic cytosolic serine/threonine catalytic protein kinase domain (PF00069), the majority of BdWAK proteins contain an extracellular galacturonan-binding (GUB) domain (PF13947) and a calcium-binding EGF-like (EGF-Ca) domain (PF07645) (Figure 1). Among the 115 WAK members, a total of 37 BdWAKs were identified as non-typical WAK structures containing either additional motifs or truncations. These include WAK proteins in class B containing a WAK family domain (PF08488), class C with a wall-associated receptor kinase C-terminal (WAK-assoc) domain (PF14380), and class D with a human EGF-Like (hEGF) domain (PF12661) [54]. Some members of the BdWAK family have truncations resulting in presence of only the kinase domain or only the extracellular domain (class G and class H). Class G proteins are still considered as members of the WAK family if the kinase domain displayed a higher similarity to known WAKs than to other plant RLKs in the BLAST database [40]. 

Phylogenetic analysis (Figure S1) of *B. distachyon* and *A. thaliana* WAK sequences demonstrated that three primary clades (1 through 3) and several sub-clades are discernible. Primary clade 1 contains 11 proteins: three AtWAKLs and eight BdWAKs (including all five class C BdWAKs), implying the proteins in this clade might have evolved from a common ancestor of dicots and grasses. While primary clade 2 contains only two AtWAKLs (AtWAKL15 and AtWAKL20), primary clade 3 contains the majority of the identified *B. distachyon* and *A. thaliana* WAKs and is comprised of two sub-clades, one minor (termed sub-clade 3A) and one major (termed sub-clade 3B). Sub-clade 3A consists of 14 proteins, all of which are AtWAKLs, while sub-clade 3B can be further divided into four sub-clades (3B-I through 3B-IV). Sub-clade 3B-I includes all five AtWAKs and two AtWAKLs, while 107 of the BdWAKs are clustered into the other major sub-clades (sub-clade 3B-II, 3B-III, 3B-IV). The largest of these subclades (3B-II) contains 54 BdWAKs, all of which display the protein structure of class A (Figure 1). The remaining class A BdWAKs are distributed throughout subclades 3B-III and 3B-IV. The phylogenetic analysis suggests that the majority of BdWAKs are distinct from those found in *A. thaliana* and emerged from expansion after the divergence of grasses and dicots. The presence of *BdWAK* gene clusters with high sequence similarity indicates that these *BdWAK* genes likely arose from localized gene duplications, as has been previously observed for *WAK* genes in rice [55].

### 3.2. Expression Profiling of B. distachyon WAK Genes

To identify *BdWAK* genes potentially involved in cell expansion, an RNA-seq analysis of *B. distachyon* coleoptiles was performed. Coleoptiles display rapid growth as a result of high rates of cell division and expansion [56]. The RNA-seq analysis was performed on *B. distachyon* coleoptiles at 48 h post-germination, a stage of rapid expansion (Figure S2). Among the highest expressed transcripts identified in this dataset are genes involved in cell wall synthesis and cell expansion, such as those encoding expansins, BURP-domain proteins, arabinogalactan-proteins (AGPs), and xyloglucan endotransglucosylases (XETs) (Table S2, Data file S1). Examination of cellulose synthase (*BdCESA*) family members showed that the primary cell wall-associated *BdCESA1*, *3*, *6*, and *9* are highly expressed compared to those associated with secondary cell wall synthesis (*BdCESA4*,*7* and *8*) or of unassigned function (*BdCESA2* and *5*) [57] (Figure S3). This is in agreement with previous qPCR studies [58] and, in conjunction with the highly expressed gene families summarised in Table S2, indicates that *B. distachyon* coleoptiles at 48 h post-germination are a suitable example of a rapidly growing tissue with cell wall synthesis and expansion occurring. 

**Figure 2:**
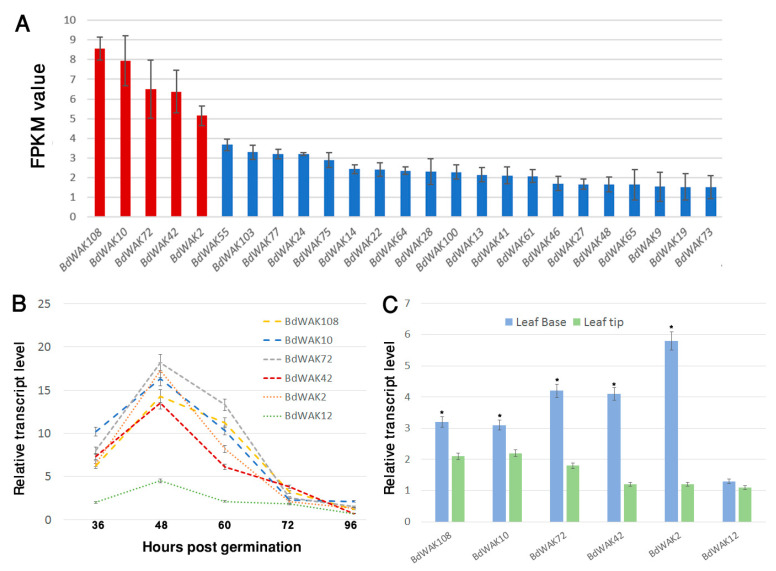
Expression analysis of *BdWAK* genes. (**A**) Transcriptomic data of *BdWAKs* arranged by descending expression (FPKM) values from *B. distachyon* coleoptiles 48 h post-germination (25 highest expressed *BdWAK*s shown). Five selected for further investigation are in red. (**B**) Expression levels of *BdWAK* genes determined via qPCR analysis of coleoptiles in a time course from 36 h to 96 h post-germination and (**C**) in leaf tissues (base and tip), normalised using reference genes listed in Table S1. Data are replicates of three biological repeats. Asterisk in (C) indicates cases where transcript level in leaf base is significantly increased compared to leaf tip (Student’s *t*-test. *p* < 0.01). Error bars indicate standard error.

The majority of *BdWAK* genes (79 out of 115 from online databases) were identified in the coleoptile transcriptome suggesting important, and possibly redundant, roles in coleoptile expansion (Figure 2A). Of the 79 *BdWAK* genes identified in the *B. distachyon* coleoptile, five members—*BdWAK2*, *BdWAK10*, *BdWAK42*, *BdWAK72*, and *BdWAK108—*were identified as the most highly expressed by mean FPKM (fragments per kilobase of transcript per million mapped reads) values (Figure 2A, Data file S1).

Except for BdWAK72 (class E), the protein product of each of these *BdWAK* genes with the highest FPKM values belongs to class A (Figure 1). qPCR analysis was performed to verify the transcriptomic data and further investigate the levels and dynamics of *BdWAK* expression during coleoptile growth. Transcript levels for the five relatively highly expressed *BdWAK* genes were investigated in *B. distachyon* coleoptiles from 36 h until 96 h post-germination. To validate the RNA-seq data, *BdWAK12* was also chosen for further analysis as it displayed relatively low FPKM values compared to the five selected *BdWAK* genes (Data file S1, sheet 2). The five *BdWAK* genes demonstrated a peak of expression at 48 h post-germination (Figure 2B), and expression rapidly decreased thereafter, coinciding with the slower growth rate of the *B. distachyon* coleoptile during this stage (Figure S2). As indicated by the transcriptomic data, *BdWAK2*, *BdWAK10*, *BdWAK42*, *BdWAK72*, and *BdWAK108* were all more highly expressed than the control *BdWAK12*, the expression of which remained relatively unchanged during coleoptile growth.

Further qPCR analysis was performed to analyse the expression of these selected *BdWAK* genes in the leaves of *B. distachyon* seedlings; in particular, leaf tips and base were examined. It was predicted that *BdWAK* genes would be more highly expressed in the leaf base if they are involved in cell expansion. Indeed, *BdWAK2*, *BdWAK10*, *BdWAK42*, *BdWAK72*, and *BdWAK108* all demonstrated a significantly higher transcript levels in the leaf base than the tip, with *BdWAK2* exhibiting the highest level of expression of the *BdWAK*s tested (Figure 2C). *BdWAK12* showed lower levels of expression in the leaf base than the other *BdWAK*s examined and displayed a similar level of expression in the leaf base and tip. 

**Figure 3:**
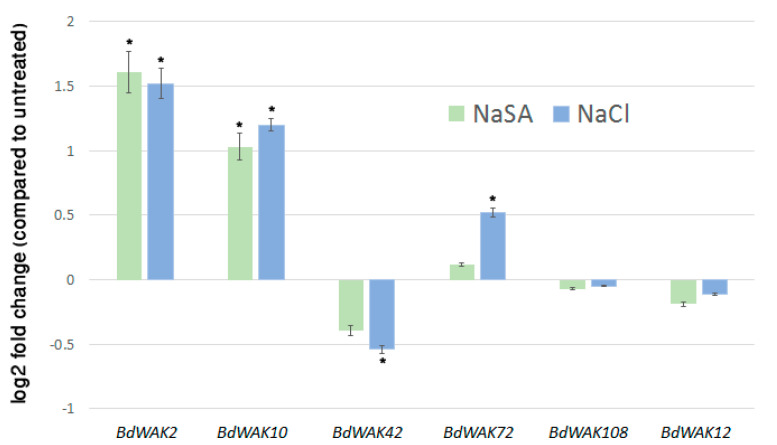
Transcript levels of selected *BdWAK* genes as determined by qPCR in *B. distachyon* seedlings in response to 0.5 mM NaSA (green) and 250 mM NaCl (blue) treatment. Data shown as log2 fold change relative to untreated control. Each measurement was performed in triplicate, with three biological replicates. Expression levels normalised to reference genes listed in Table S1. Asterisk indicates ∆CT values that were significantly different between treated (NaSA or NaCl) and untreated sample (Student’s *t*-test, *p* < 0.05). Error bars indicate standard error.

In addition to regulating cell expansion, *WAK* genes have also been shown to be responsive to various environmental stresses [21,23,59]. In order to determine if any of the *BdWAK* genes implicated in cell wall expansion are also involved in response to external stresses, 0.5 mM NaSA and 250 mM sodium chloride (NaCl) were applied to hydroponically growing *B. distachyon* seedlings. Expression of the selected five *BdWAK* genes was examined three days after the initiation of the treatment. Two *BdWAKs* (*BdWAK2* and *BdWAK10*) displayed increased expression levels (*p* < 0.01 and *p* < 0.05, respectively) in response to the NaSA treatment (Figure 3), which induces defence response. In response to salt treatment, *BdWAK2* and *BdWAK10* showed significantly (*p* < 0.01) increased expression levels, while *BdWAK72* showed a less, but still significant (*p* < 0.05) increase in transcript levels. *BdWAK42* displayed reduced expression in response to NaCl (*p* < 0.05), but *BdWAK108* and *BdWAK12* showed no significant change in expression in response to either treatment. 

### 3.3. BdWAK Extracellular Domain In Vitro Binding Assay

In *A. thaliana*, WAKs have been identified as pectin-binding proteins [16,17], and this binding may be essential for signal transduction function. In order to explore this possibility in grasses, the polysaccharide binding properties of various BdWAKs were examined. BdWAK extracellular domains fused to a 6× His tag were expressed in *E. coli* and enriched using affinity chromatography (Figure S4). Using an ELISA-based binding assay [18], various polysaccharides were tested for binding capability with the extracellular domain of candidate BdWAKs (BdWAK2, BdWAK10, BdWAK42, BdWAK72, BdWAK108, and control BdWAK12) and a positive control, AtWAK2, previously shown to bind a mixed-length pectin polymer purified from citrus [60].

**Figure 4:**
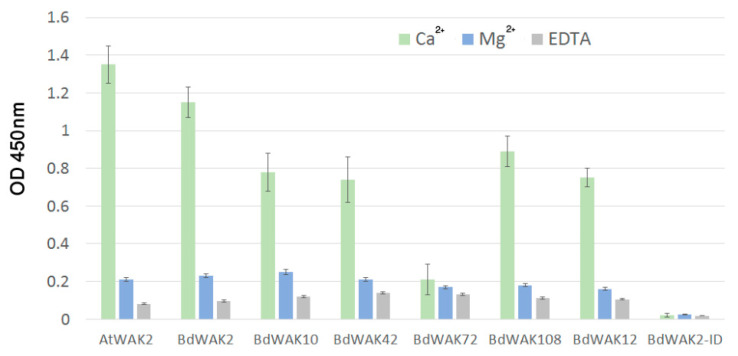
ELISA assay for interaction of recombinant WAK extracellular domains with PGA. PGA prepared in 0.5 mM Ca^2+^/150 mM Na^+^ Tris buffer (green), in 0.5 mM Mg^2+^/150 mM Na^+^ Tris buffer in which Ca^2+^ is replaced by Mg^2+^ (blue) or in 0.5 mM Ca^2+^/150 mM Na^+^ Tris buffer + 5 mM EDTA (grey) was used to coat ELISA plates. BdWAK2-ID is the intracellular domain of BdWAK2 included as a negative control. The presence of WAK extracellular domain remaining bound to PGA was detected by anti-6× His antibody binding (and subsequent application of secondary mouse-HRP antibody) to the His-tagged extracellular domain, and quantified by the colour change of TMB (a HRP substrate) at OD 450 nm. Each measurement was performed in triplicate. Error bars indicate standard error.

Previous experiments have shown that an ionic environment with the presence of Ca^2+^ (0.5 mM Ca^2+^/150 mM Na^+^ Tris buffer) promotes the formation of polygalacturonic acid (PGA) dimers [18,61] that both enhance gelling (i.e., increases the viscosity of the cell walls gel-like matrix) and allow for binding of WAK extracellular domain to PGA. All BdWAK extracellular domains tested, with the exception of BdWAK72, showed strong interaction with PGA (Figure 4). The intracellular domain of BdWAK2 (BdAWAK2-ID) did not show interaction with PGA. To examine whether the interactions observed where dependent on Ca^2+^ ionic conditions promoting PGA dimers, interactions were tested in an environment where calcium was either replaced with magnesium (Mg^2+^/150 mM Na^+^ Tris buffer) or where ethylenediaminetetraacetic acid (EDTA) was added to the calcium buffer (where EDTA chelates calcium and is proposed to inhibit formation of PGA dimers) [18]. In these conditions, binding of the various WAK extracellular domains to PGA was greatly reduced (Figure 4), indicating that the conformation of PGA influences the binding to BdWAK extracellular domains in a similar manner observed previously for AtWAKs [60]. 

**Figure 5:**
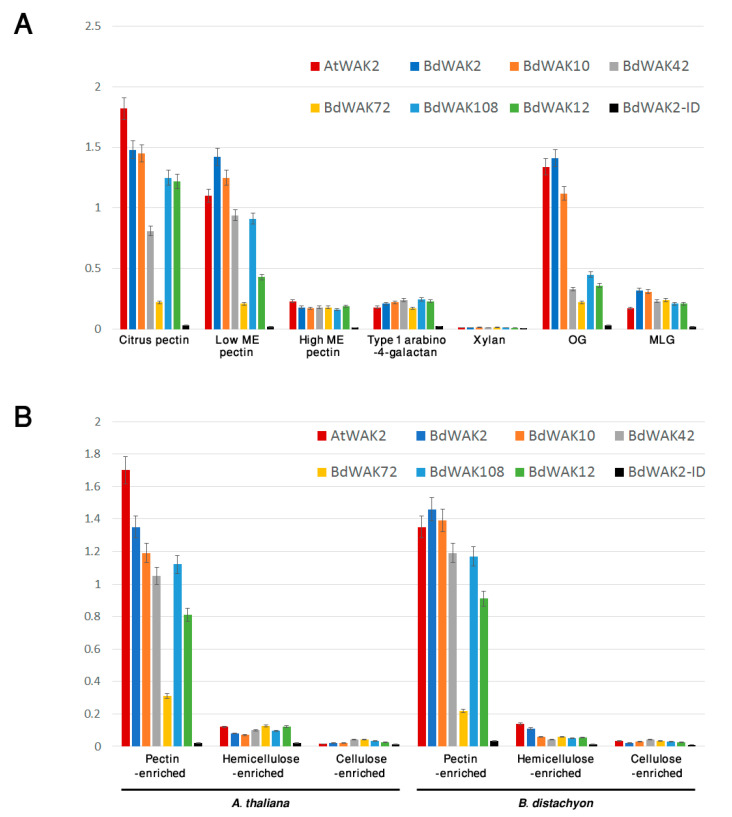
(**A**) ELISA assay for interaction of recombinant WAK extracellular domains with various cell wall polysaccharides. Results are expressed as conversion of TMB substrate at OD 450 nm which measures the presence of WAK extracellular domain remaining bound to coated polysaccharide using antibody detection of His-tagged recombinant proteins. Each measurement was performed in triplicate. ME = methyl-esterified. MLG = Mixed-linkage glucan. OG = oligogalacturonides. (**B**) ELISA assay for interaction of recombinant WAK extracellular domains with either *A. thaliana* or *B. distachyon* wall fractions enriched for pectin, hemicellulose, or cellulose. Error bars indicate standard error.

Binding of the candidate BdWAK extracellular domains to a range of cell wall polysaccharides was subsequently examined. Except for BdWAK72, all extracellular BdWAK domains tested showed binding with citrus pectin (galacturonic acid, ≥74.0%) and lowly methyl-esterified pectin, but not to either highly methyl-esterified pectin, type I arabino-4-galactan or xylan (Figure 5A). Binding to OG (DP > 9) was also examined, where AtWAK2 and BdWAK2 showed the strongest binding, followed by BdWAK10. Other BdWAKs (BdWAK42, BdWAK72, and BdWAK108) showed much lower binding affinity for OGs. Binding to mixed-linkage glucan (MLG, extracted from barley), absent in *A. thaliana*, was at a low level and not significantly different between BdWAKs and AtWAK2. 

Walls isolated from fresh leaves of *B. distachyon* and *A. thaliana* and enriched for different polysaccharide classes via chemical fractionation were examined for interaction with BdWAK extracellular domains. All WAK extracellular domains except for BdWAK72 showed strong interaction with the pectin-enriched wall extracts and little binding affinity to either hemicellulose- or cellulose-enriched fractions from either *A. thaliana* or *B. distachyon* (Figure 5B). Protein alignment of the extracellular domain of the tested BdWAK candidates (Figure S5) reveals that while BdWAK72 contains a canonical GUB domain, there are several amino acid regions where BdWAK72 differs (Figure S5, blue underlined) from the other BdWAKs tested, which may provide some insight into key regions for mutagenesis studies to more precisely define BdWAK binding to pectin. AtWAK2 demonstrated a stronger binding affinity for the *A. thaliana* pectin-enriched wall fraction compared to the BdWAKs and possessed a similar affinity for *B. distachyon* pectin fractions as the strongest-binding BdWAKs extracellular domains, such as BdWAK2 and BdWAK10 (Figure 5B).

*BdWAK2* was selected for further investigation in (a) biotic stress interactions as it displayed relatively high expression in expanding tissues of seedlings, increased expression in response to external stresses and the strongest binding to pectin.

### 3.4. BdWAK2 Triggers Cell Death in N. benthamiana Leaves

**Figure 6:**
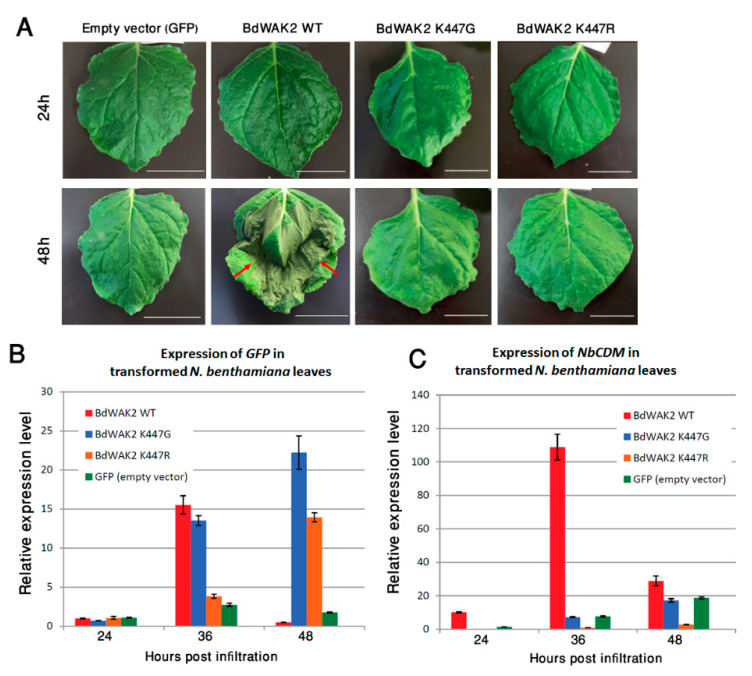
Cell death response in *N. benthamiana* leaves infiltrated with BdWAK2 kinase active and inactive variants. (**A**) *N. benthamiana* leaves with constructs containing the Ubiquitin10 promoter driving GFP only (Empty vector), wild-type BdWAK2 (BdWAK2 WT) and mutated BdWAK2 (K447G and K447R), are shown 24 h and 48 h post-*Agrobacterium* infiltration. Cell death as evidenced by necrotic lesions (indicated by red arrows) was visible on the leaf transformed with BdWAK2 WT after 48h, but not on the leaves transformed with empty vector or mutated BdWAK2. Scale bar = 20 mm. *n* ≥ 3 leaves per construct. Transcript levels of *GFP* (**B**) and cell death marker gene *NbCDM* (**C**) as determined by qPCR in *N. benthamiana* leaves infiltrated with the constructs described in (**A**). Expression levels are normalised with reference genes listed in Table S1. Data are replicates of three biological repeats. Error bars indicate standard error.

WAKs have previously been shown to play roles in plant defence; the response at the transcriptional level of *BdWAK2* to external stresses suggests that it may play a role in biotic or abiotic stress responses. To further characterize BdWAK2, the protein sequence was tagged with a C-terminal green fluorescent protein (GFP) and transiently expressed in *N. benthamiana* leaves via *Agrobacterium* infiltration. Subcellular localization of BdWAK2 was unable to be observed due to a necrotic-like lesion phenotype which was observed as early as 36 h post-*Agrobacterium* infiltration, with the infiltrated area of the leaves demonstrating cell death by 48 h (Figure 6A). Infiltration of *N. benthamiana* leaves with either the empty expression vector, the *Agrobacterium* strain (untransformed), or the *Agrobacterium* transformation enhancer line P19 did not result in a similar cell death phenotype. This pointed to a possible role of BdWAK2 as part of the plant’s hypersensitive immune response. 

To assess whether the kinase activity of BdWAK2 was responsible for the observed cell death in *N. benthamiana* leaves, mutations were introduced into a conserved catalytic amino acid (K447) in the kinase domain of BdWAK2 to eliminate kinase activity. This invariant lysine (K) is crucial for kinase activity due to its involvement of anchoring and orienting ATP for the process of phosphorylation [62,63]. The mutated BdWAK2 kinase domain (either K447G or K447R), the WT BdWAK2 kinase domain, and the kinase domain of AtBRI1 (Brassinosteroid Insensitive 1; a known active kinase) were expressed in *E. coli* (BL21) and tested for kinase activity. Examination of these expressed kinase domains via Pro-Q staining showed that the kinase activity of the mutated BdWAK2 was reduced by approximately 75% (K447R) and 88% (K447G) relative to WT BdWAK2, respectively (Figure S6).

When full-length BdWAK2 proteins with an active site mutation (either K447G or K447R) were expressed in *N. benthamiana* leaves, cell death at 48 h post-infiltration was not observed, whereas the full-length WT BdWAK2 caused necrotic lesions and cell death (Figure 6A). Infiltrated leaves transformed with the mutated versions of BdWAK2 did not display any obvious necrosis even after 7 days (data not shown). To confirm that the mutated forms of BdWAK2 fused to GFP were being expressed in *N. benthamiana* leaves, expression levels of GFP was determined by qPCR. Similar levels of *GFP* transcripts were detected in *N. benthamiana* leaves for BdWAK2 kinase active and inactive constructs (Figure 6B). The expression of the cell death marker gene *NbCDM* [64] in transformed leaf samples was examined via qPCR at 24 h, 36 h, and 48 h post-infiltration. At 24 h post-infiltration, the expression of *NbCDM* was at a low level in all samples (Figure 6C). In leaves transformed with WT BdWAK2 the expression level of *NbCDM* increased dramatically (approximately 10-fold) at 36 h post-infiltration compared to leaves transformed with mutated BdWAK2, and this expression decreased at 48 h post-infiltration, likely due to the widespread cell death occurring in these leaves at this stage. Only the WT BdWAK2 with an active kinase domain was able to induce cell death upon over-expression in *N. benthamiana* leaves and inactivating the BdWAK2 kinase domain eliminated these deleterious effects.

## 4. Discussion

The WAK family of RLKs are strong candidates for cell wall-related signaling in response to developmental and environmental stresses in order to maintain cell wall integrity. This study shows that WAKs in *B. distachyon* can interact with cell wall pectins and are likely to play roles in cell expansion and stress responses. Similar to other grass species, there are a large number of WAK gene family members in *B. distachyon*. There are 125 WAKs in rice [55], more than 100 WAKs in maize [27], 91 WAKs in barley [65] and 115 WAKs in *B. distachyon* compared to only 26 members in *A. thaliana*. A typical WAK protein in *A. thaliana* contains a GUB domain, an EGF-Ca domain, and a protein kinase domain [16]. Although high diversity of extracellular domains seems to be a common feature of plant WAKs, some BdWAKs do not contain either the EGF2-like (PS01186) or the EGF3-like (PS50026) domains observed in both *A. thaliana* [19] and *Populus* [66]. The presence of the EGF-like domain implies potential protein-protein interactions [67], whereas the detailed functions of the various extracellular motifs identified in this study, such as WAK Family and WAK-assoc domains, remains unclear. It may be notable that the extracellular domain of BdWAK72 showed much lower pectin binding capability than the other BdWAKs tested, and it is also the only one that falls outside of structural class A (Figure 1). Previous studies [18,24] identified a region in AtWAK1 (aa 67-245) responsible for pectin binding. Two of the regions in which BdWAK72 differs from the other BdWAKs fall within this range (Figure S5). Further functional analysis of these regions could illuminate the key residues required for WAK-pectin binding.

Coleoptiles undergo rapid growth soon after seed germination, making them an ideal system for the study of cell expansion in commelinid monocots, that include the grasses. RNA-seq analysis of *B. distachyon* coleoptiles at 48 h post-germination identified the most highly expressed *BdWAK*s. Investigation of the expression dynamics of these *BdWAK*s showed peak expression at the stage with the most rapid coleoptile growth (48 h post-germination), suggesting they may be involved in receiving signals as part of the cell expansion mechanism. Promoter::GUS analysis of the WAK *OsDEES1* (structural class A) in rice [30] also demonstrated strong expression in the coleoptile. Although our transcriptomic analysis of 48 h post-germination coleoptiles has aided the identification of the highest expressed *BdWAK*s amongst a large gene family, it is probable that other lower-expressed *BdWAK* genes are also participating in the cell expansion process.

Coleoptile growth involves extensive cell wall changes to accommodate irreversible cell wall expansion, thus many cell wall-related genes are upregulated during its growth. For example, barley XET genes (xyloglucan endo-transglucosylases), which mediate the cleavage and reconnection of the β-(1-4)-glucan backbone of XGs of the primary cell wall, demonstrate an expression pattern correlated to coleoptile growth [68]. In this study several *B. distachyon* genes encoding putative XETs and expansins were found to be highly expressed at the peak coleoptile growth stage (Table S2, Data file S1). The expansion of primary cell walls is driven by turgor pressure [4,69], and the *BdWAKs* expressed in the coleoptile may be involved in the regulation of turgor pressure by activating the expression of vacuolar invertase genes, as has been demonstrated in *A. thaliana* [70]. Indeed, a single *B. distachyon* vacuolar invertase gene was identified in the coleoptile transcriptome (Bradi1g52210) and it was among the highest expressed transcripts (top 0.05 percentile) at this stage of rapid coleoptile growth.

Plants experience both biotic and abiotic stresses and have acquired sophisticated response mechanisms to cope in these environments. The expression of *AtWAKs* is induced under biotic stresses such as a pathogen response [20,21], as well as abiotic stresses such as either physical wounding or mineral toxicity [17,22,59]. In our search for *BdWAK* genes involved in both cell expansion and stress responses, those which were found to be most highly expressed during rapid coleoptile expansion were subsequently examined under NaSA-induced (to mimic a biotic stress) and salt stress (an exemplar abiotic stress), revealing that a subset of the examined *BdWAK* genes displayed increased expression under either one or both types of stress conditions. *BdWAK2* and *BdWAK10* displayed a significant increase in expression levels in response to both NaSA and salt treatments, whereas *BdWAK72* was induced only in response to salt stress. Given that *BdWAK2* and *BdWAK10* are inducible under multiple types of stresses (biotic and abiotic), it is possible that they sense a general stimulus derived from both biotic and abiotic stresses. In support of this, a previous report of a transcriptomic analysis in *B. distachyon* to investigate gene expression response to various phytohormones found that *BdWAK2* was the mostly highly upregulated among the three *BdWAK* genes found to be responsive to jasmonic acid [71].

Transient overexpression of BdWAK2 (WT) in *N. benthamiana* leaves induced severe necrosis. Protein kinases involved in signaling cascades are often linked to programmed cell death (PCD; apoptosis) [72,73]. Many Ser/Thr kinases, such as MAPKs play roles in apoptosis [74] and it is known that AtWAKs activate MAPK pathways [60,75]. It is possible that over-expression of BdWAK2 mimics a hypersensitive response to pathogen challenge, resulting in cell death. Similarly, a recent study demonstrated heterologous expression of a rice *WAKL* gene in *A. thaliana* (OsWAKL21.2) can activate plant immune response [76]. ZmWAK-RLK1, encoded by the *Htn1* gene [77] was found to confer resistance to northern corn leaf blight by reducing benzoxazinoid secondary metabolites [78]. A study of two near-isogenic lines in maize [37] found that a wall-associated kinase termed ZmWAK was responsible for acute localised cell death in response to *Sphacelotheca reiliana* attack. BdWAK2 may respond to pathogen challenge in a similar manner given the results observed in *N. benthamiana*. While non-RD WAKs (which includes BdWAK2), have generally been described as participating in response to pathogens [32], this study indicates that the binding capacity of the extracellular domain may also play a role in this process.

The role of phosphorylation is an emerging interest in WAK signaling. In a large-scale phosphoproteome analysis of seedling leaves in *B. distachyon*, a single WAK (BdWAK10) was identified as one of 950 phosphoproteins [79]. In the present study, we found BdWAK10 has potential roles in both cell expansion and stress response. BdWAK10 was found to be phosphorylated at the Ser693 position of the intracellular kinase domain, indicating potential auto-phosphorylation as part of a self-regulatory mechanism. Phosphoproteomic analysis in *A. thaliana* showed that signaling pathways that lead to phosphorylation of proteins due to exposure to OGs are largely distinct from other signaling pathways [53]. Determining the conditions under which BdWAK10 and other BdWAKs become phosphorylated would provide insight into their regulatory mechanisms/pathways.

As WAKs are proposed to perceive extracellular stimuli and mediate intercellular signaling pathways, it is possible that some *WAK* genes are upregulated due to changes in the extracellular matrix under stress conditions. In our investigation, the extracellular domains of BdWAK proteins were shown to be able to bind with pectins and not with other wall-associated polysaccharides. BdWAK-pectin binding was found to be dependent on pectins forming inter-molecular bridges in the presence of Ca^2+^. Pectins are involved in many physiological processes such as cell growth control and signaling [10,80,81,82]. A previously proposed model for WAK signaling suggests that the nature of the binding between WAKs and pectins could be important for determining which downstream pathways are activated in response to the existing environmental conditions, such as the presence or absence of stresses [83]. For example, pectins are able to form a flexible matrix that allows modifications when plant cell walls undergo structural changes, and these changes can be induced by either developmental signals (i.e., cell expansion which requires reconstruction of cell walls) or defence signals (i.e., pathogen release of wall degrading enzymes) [81,82]. Therefore, the binding between WAKs and pectins may be essential for the regulation of cell expansion and/or defence responses. Our results show that, like AtWAK2, the extracellular domain of some BdWAKs (such as BdWAK2 and BdWAK10) bind to native pectins in addition to OGs. Although the pectin content of grass cell walls is comparatively low, our in vitro assays show that BdWAKs still possess a strong binding affinity for pectins. This suggests that even a low quantity of pectin in the cell wall is enough to allow for proper binding of WAK extracellular domain to the cell wall and potentially initiate downstream signaling. No other wall-associated polysaccharide binding was observed for BdWAKs, although not all cell wall polysaccharides were tested in this study. The fact that *BdWAK72* displayed relatively high expression level in the developing coleoptile and showed sensitivity to salt stress, but exhibited low binding capability to pectins suggests a possible divergence in the mechanism by which BdWAK72 perceives changes in the cell. It is possible that the proteins products of *BdWAK* genes with similar expression profiles might form heterodimers (as has previously been shown in rice [84]), in which case not all BdWAKs would be required to fulfil the role of pectin binding. While AtWAK2 showed the strongest binding affinity (compared to the tested BdWAKs) to pectin-enriched cell wall extracts derived from *A. thaliana*, the binding of AtWAK2 and BdWAKs to pectin-enriched *B. distachyon* cell walls was similar. Further analysis is required to translate these results to the *in planta* situation, where differences in the quantity of pectin present in *B. distachyon* tissues compared to *A. thaliana* [34] may alter the ability of BdWAK sub-classes to interact with cell walls.

We observed relatively high expression levels of *BdWAK2* and *BdWAK10* both in expanding tissues and under stress (both NaSA and salt treatment). While this appears to present a contradiction, this pattern of gene expression may be consistent with a signaling model where, depending on the conformation of pectin present (pectin polymers in the native cell wall, or OG fragments generated under stress conditions), different downstream pathways are triggered by a given WAK [12]. Our in-vitro analysis shows that BdWAK2 and BdWAK10 can bind both pectic polysaccharides extracted from the cell wall and shorter OG fragments derived from pectic polysaccharides, and that distinct signaling cascades may be triggered upon reception of each. It is notable that while expression of *BdWAK72* was induced under salt stress, it was not induced in response to NaSA, nor did it show binding to OGs, suggesting that the increase in *BdWAK72* expression in response to salt stress may be due to cell wall modifications that occur specifically during salt stress.

## 5. Conclusions

In this study we have analysed the family of *WAK* genes in *B. distachyon*. A transcriptomics approach was utilized to identify those *BdWAK*s expressed in the expanding coleoptile, among which *BdWAK2*appears to be involved in multiple signaling pathways as evident by increased expression in response to NaSA and salt treatments, and the induction of cell death when over-expressed in *N. benthamiana*. The BdWAK2 extracellular domain binds pectin in a similar manner to that observed in dicots, despite the low levels of pectin in the cell walls of grass species. The varying pectin-binding capabilities of the extracellular domains of several BdWAKs has aided in identifying amino acid regions that may influence the interaction between grass WAKs and pectin. Analysis of mutant or over-expression lines of these identified candidate *WAK*s in *B. distachyon* would allow a greater understanding of the relationship between their functional role and their binding capabilities.

## Supplementary Materials

The following are available online at https://www.mdpi.com/2073-4409/9/11/2478/s1, Figure S1: Phylogenetic analysis showing the relationships between the five *A. thaliana* WAKs (AtWAKs), 21 *A. thaliana* WAK-Like (AtWAKLs), and 115 *B. distachyon* WAKs (BdWAKs). Figure S2: The average daily growth rate of *B. distachyon* coleoptiles from the beginning of germination to 96 h post-germination. Figure S3: RNA-seq analysis showing *BdCESA* gene expression levels in expanding *B. distachyon* coleoptiles at 48 h post germination. Figure S4: Coomassie stained SDS gel showing affinity purified recombinant proteins used in binding assay. Figure S5: Amino acid alignment of the extracellular domain of AtWAK2, BdWAK2, BdWAK10, BdWAK42, BdWAK72 and BdWAK108. Figure S6: Relative kinase activity of BdWAK2 variants and AtBRI1. Table S1: Primers used in this study. Table S2: Cell wall protein families encoded by genes highly expressed in *B. distachyon* coleoptile transcriptomic data at 48 h post-germination. Data file S1: Summary of RNA-seq data file. Sheet 1: List of 100 highest expressed transcripts identified in *B. distachyon* coleoptile RNA-seq analysis (based on mean FPKM values from three replicates). Sheet 2: List of 79 *BdWAK* transcripts identified in RNA-seq analysis of *B. distachyon* coleoptile.
