# Supplementary material for: The Role of Brachypodium distachyon Wall-Associated Kinases (WAKs) in Cell Expansion and Stress Responses
Source: Cells. 2020 Nov 14;9(11):2478. doi: 10.3390/cells9112478 (PMC7698158; doi:10.3390/cells9112478)
Supplement: Supplementary file 1 [file cells-09-02478-s001.zip › Supplementary data plus legends.pdf]

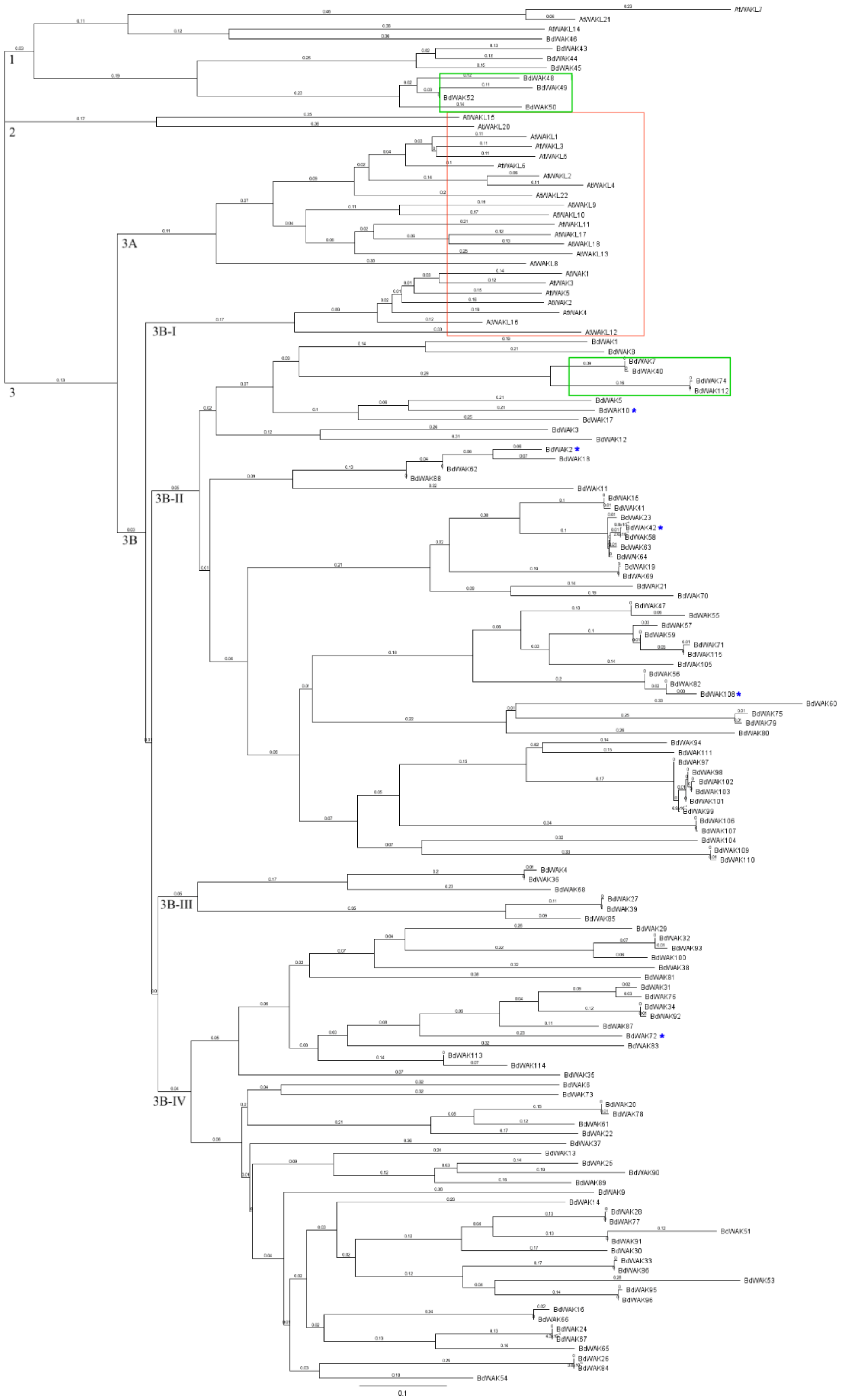

Figure S1. Phylogenetic analysis showing the relationships between the five ~~*Arabidopsis*~~ *A. thaliana* WAKs (AtWAKs), 21 ~~*Arabidopsis*~~ *A. thaliana* WAK-Like (AtWAKLs), and 115 ~~*Brachypodium*~~ *B. distachyon* WAKs~~WAK/Ls (~~(BdWAKs~~)~~). The tree was generated using the Geneious ver 5.6.6 (Kearse et al. 2012) software based on full length protein sequences of WAKs using the Neighbor-Joining method (Saitou and Nei 1987). The genetic distance was calculated using the Jukes-Cantor

Model (Jukes and Cantor 1969) and branch labels indicate changes in amino acids (substitutions per site). Clades and sub-clades are indicated as: Digits (1 through 3) for the major clades; Capital letters (A and B) for the sub-clades of major clade 3; Roman numerals (I through IV) for the sub-clades of clade 3B. The clades consisting exclusively of AtWAKs and AtWAKLs are outlined in red rectangle. Scale bar = 0.1 amino acid substitutions per site. Blue asterisks indicate BdWAKs examined further in this study. Green rectangles indicate BdWAKs which are physically clustered on *B. distachyon* chromosome 2 and demonstrate high sequence similarity. Accession numbers listed in Data file S1.

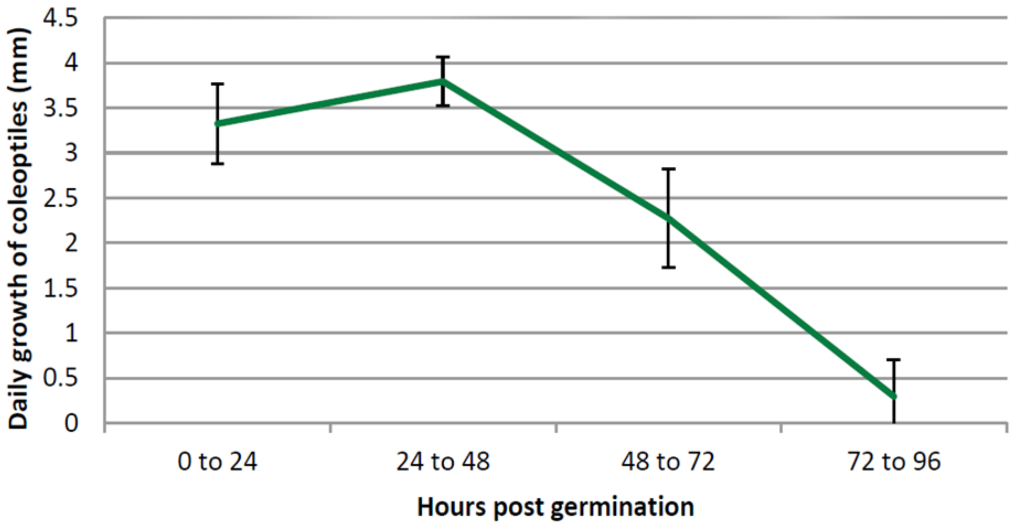

Figure S2. The average daily growth rate of ~~*Brachypodium*~~ *B. distachyon* coleoptiles from the beginning of germination to 96 h post-germination. The growth rate is presented in length (mm) and calculated as the average length of coleoptiles minus the average length 24 h before. Standard error bar is shown.

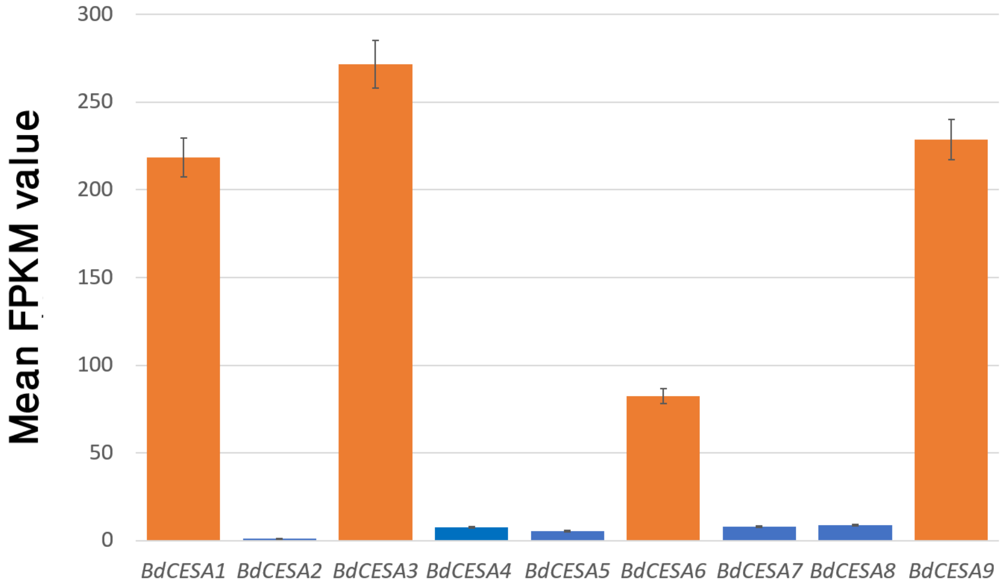

Figure S3. RNA-seq analysis showing *BdCESA* gene expression levels in expanding ~~*Brachypodium*~~ *B. distachyon* coleoptiles at 48 h post germination (mean FPKM value). *BdCESA* genes associated with primary cell wall synthesis (orange) were highly expressed compared with those associated with either secondary wall synthesis or with unassigned function (blue). Error bars indicate standard error. Each measurement was performed with three biological replicates. Sequence accession numbers: *BdCESA1* (Bradi2g34240), *BdCESA2* (Bradi1g04597), *BdCESA3* (Bradi1g54250), *BdCESA4* (Bradi3g28350), *BdCESA5* (Bradi1g29060), *BdCESA6* (Bradi1g53207), *BdCESA7* (Bradi4g30540), *BdCESA8* (Bradi2g49912), *BdCESA9* (Bradi1g02510).

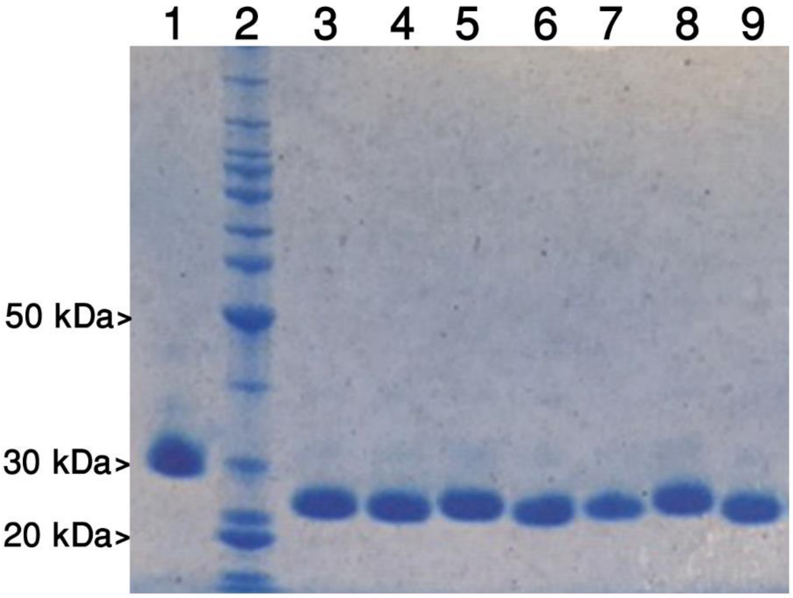

Figure S4. Coomassie stained SDS gel showing affinity purified recombinant proteins used in binding assay. BdWAK2 intracellular domain (lane 1), AtWAK2 extracellular domain (EXD) (lane 3), BdWAK2 EXD (lane 4), BdWAK10 EXD (lane 5), BdWAK12 EXD (lane 6), BdWAK42 EXD (lane 7), BdWAK72 EXD (lane 8), BdWAK108 EXD (lane 9). Molecular weight indicated by Benchmark™ Unstained Protein Ladder (Novex) (lane 2).

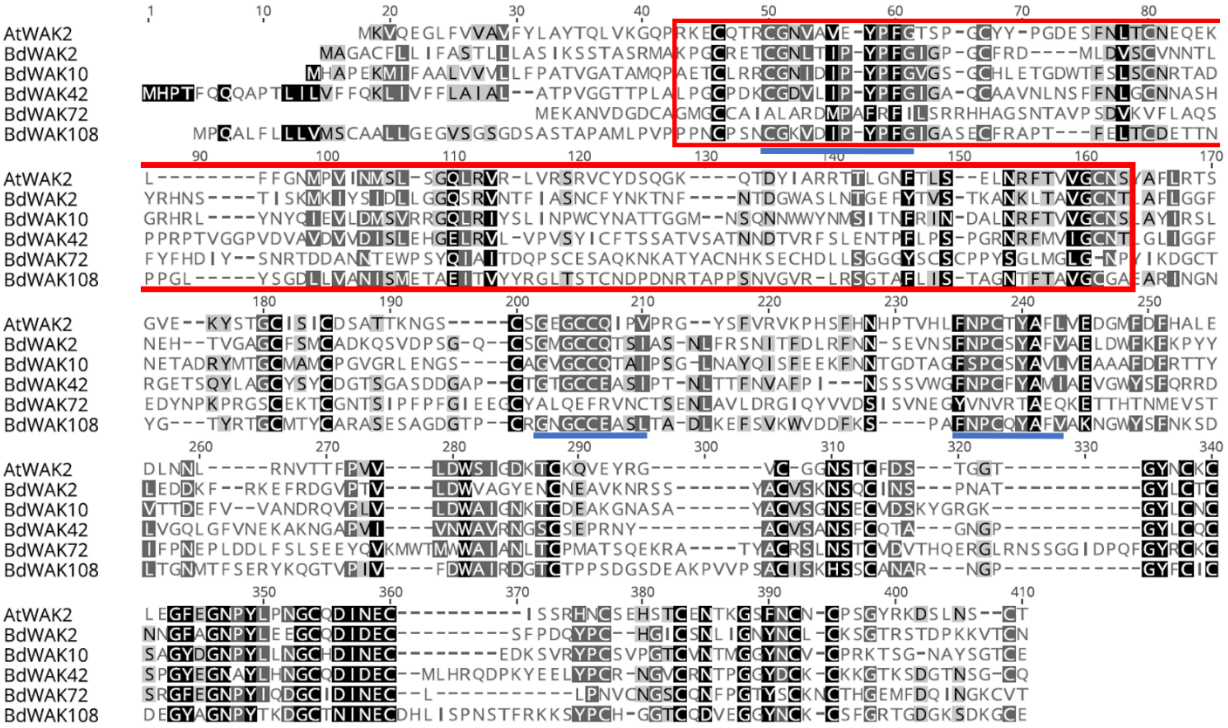

Figure ~~S4~~S5. Amino acid alignment of the extracellular domain of AtWAK2, BdWAK2, BdWAK10, BdWAK42, BdWAK72 and BdWAK108. The galacturonan-binding domain (GUB) is outlined by a red square. Regions in which BdWAK72 appears distinct from all other BdWAKs are underlined in blue.

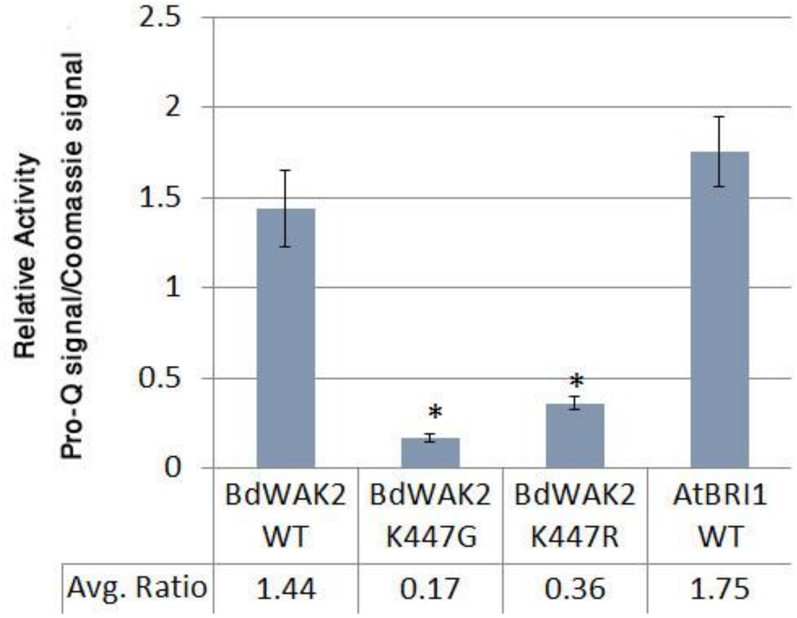

Figure ~~S5~~S6. Relative kinase activity of BdWAK2 variants and AtBRI1. Data is presented as the average ratio of Pro-Q staining to Coomassie staining signal strength of non-mutated BdWAK2 (WT), two versions of BdWAK2 with point mutations in the invariant lysine (K) crucial for kinase activity (K447G and K447R), and AtBRI1 (positive control). A statistically significant reduction in kinase activity compared to WT is marked with an asterisk (Student's T-test, p ~~Value~~< 0.01).

Table S1. Primers used in this study

Primers for qPCR		
BdWAK2	AGCTGGGTGTTTCTCGATGT	F
	GTTGGAGCGAAAGAGGTTTG	R
BdWAK10	TACCATGGGCGGATACAACT	F
	TGATGACACCAACGCTGACT	R
BdWAK12	AACTGCTCCAAGGGCTACAA	F
	CTTTGAGTTGGCACTCGTCA	R
BdWAK42	TGCATTCAAAAACCAATACCC	F

	CAGTTAGCAGGCCTTCCTTC	R
BdWAK72	GCACTTCAGCAGGGAGTTCT	F
	GCATGACGCATAACATGAGC	R
BdWAK108	GGAGGAACATGCCAAGATGT	F
	TTCTCTGACGATCCATGTGC	R
GFP	CAGAAGAACGGCATCAAGGT	F
	CTGGGTGCTCAGGTAGTGGT	R
~~Nb630~~NbCDM	AGCACTGGCGGAATTAAGG	F
	GAACACCCTGTAGCTCTTGGAC	R
NbActin	ATCGGAATGGAAGCTGCTGG	F
	ATCCTATCAGCAATGCCCGG	R
NbUbiquitin	CAGCTCGAGGATGGAAGGAC	F
	GACGGAGGACAAGGTGAAGG	R
BdRAR1	CCTACAAGGAGAAGGATAACC	F
	ACATGAATATCACAACATTTCC	R
BdRD22	GTGACGTTACCGTGGCATTC	F
	GCGTCGTCGTAGCAAGAGAG	R
BdELF	CCATCGATATTGCCTTGTGG	F
	GTCTGGCCATCCTTGGAGAT	R
BdGAP	TTGCTCTCCAGAGCGATGAC	F
	CTCCACGACATAATCGGCAC	R
BdTubulin	ACCAACCTTGTGCCCTATCC	F
	GGGCACCAGTCAACAAACTG	R
Primers for cloning		
BdWAK2 Kinase	GGCATCATGTCAAAACAT	F
	TCTACCACTCTCTAAATCGAG	R
AtBRI1 Kinase	AATGGTTTCCATAATGATAG	F
	TAATTTTCCTTCAGGAACTTCT	R
BdWAK2 EXD	ATGGCTGGAGCCTGC	F
	TCAGCAGGTTACTTTCTTTG	R
BdWAK10 EXD	ATGCATGCCCCAGAAAAGAT	F
	TCACCCGCTGTACGCGTTGCCT	R
BdWAK12 EXD	ATGGCTGGAGCCTGCTTCCT	F
	TCACCGGCAACCATTCTTGATG	R
BdWAK42 EXD	ATGGCCACTGCGCCCTTGCA	F
	TCAGCAGGTATCTGCTTTGCC	R
BdWAK72 EXD	ATGCCGGCATTCAGGTTCAT	F
	TCAACACATTTTCCATTTATTTG	R
BdWAK108 EXD	ATGTCATGTGCCGCTCTCCT	F
	TCAACAACCTTTGTCACTTTT	R
AtWAK2 EXD	ATGAAGGTACAGGAGGGTT	F
	TCAACGAGTACAGCTATTAAGG	R
BdWAK2 (K447G)	GTAGCAATAGGGCGCTCAAAG	F
	CTTTGAGCGCCTTATTGCTAC	R
BdWAK2 (K447R)	GTAGCAATAAGGCGCTCAAAG	F
	CTTTGAGCGCCTTATTGCTAC	R

BdWAK2 ID	GGCATCATGTCAAAACAT	F
	TCTACCACTCTCTAAATCGAG	R

Table S2. Cell wall protein families encoded by genes highly expressed in ~~*Brachypodium*~~*B. distachyon* coleoptile transcriptomic data at 48 h post-germination.
[a] Median expression level for each family is displayed as ranking percentile among expression level (FPKM values) of all transcripts identified (n=41,238)

Protein family	Role	Gene accession	Mean FPKM value	St Dev	Median expression level[a]
Xyloglucan Endotransglucosylases / Endohydrolases (XETs/XEHs)	Loosening of plant cell walls	Bradi4g16990.1	1257.84	188.43	96.1%
		Bradi1g33810.1	858.81	65.72	
		Bradi3g18600.1	676.00	61.68	
		Bradi1g33840.1	396.08	37.96	
		Bradi3g18590.1	388.67	127.89	
		Bradi3g52307.1	124.12	14.62	
		Bradi1g25847.1	106.06	14.48	
		Bradi5g22907.1	95.69	35.39	
		Bradi1g27867.1	89.77	49.74	
		Bradi1g68590.1	57.60	16.78	
		Bradi4g29707.1	41.30	21.28	
		Bradi1g33827.1	14.59	3.60	
		Bradi3g34227.1	12.81	2.12	
		Bradi1g33817.1	10.36	5.61	
		Bradi1g77990.1	4.03	1.07	
		Bradi3g10290.1	2.56	1.94	
		Bradi1g01150.1	1.095	0.38	
BURP-domain protein	Cell wall relaxation, cell expansion	Bradi1g49322.2	8318.86	1094.39	95.2%
		Bradi4g44496.1	4382.37	618.19	
		Bradi2g49000.3	139.68	47.08	
		Bradi1g50390.2	14.46	1.90	
		Bradi3g34950.1	13.27	0.82	
		Bradi1g42970.1	0.76	0.52	
Arabinogalactan-proteins (AGPs)	Cell wall remodelling, secondary wall deposition	Bradi2g16560.3	854.24	80.39	88.2%
		Bradi2g46120.1	752.77	66.29	
		Bradi2g16570.1	360.42	60.77	
		Bradi4g34420.1	326.54	27.96	

		Bradi3g39740.1	103.88	11.21	
		Bradi1g57040.1	77.79	15.93	
		Bradi1g06290.1	74.13	8.60	
		Bradi1g76630.1	39.63	6.15	
		Bradi2g23270.2	38.09	1.60	
		Bradi1g39290.1	35.84	7.08	
		Bradi2g31980.1	35.81	5.75	
		Bradi3g39010.1	31.56	2.84	
		Bradi2g54620.2	30.83	1.90	
		Bradi2g00220.1	18.33	3.19	
		Bradi4g33490.1	9.59	4.32	
		Bradi5g10985.1	10.73	0.74	
		Bradi2g34650.1	10.69	2.09	
		Bradi4g24974.1	9.36	5.97	
		Bradi4g33040.1	9.16	2.13	
		Bradi5g18950.1	6.41	2.47	
		Bradi3g15283.5	1.74	0.39	
		Bradi4g33490.1	1.11	0.35	
		Bradi4g25601.1	0.74	0.17	
		Bradi3g56390.1	0.59	0.23	
Glycine-rich cell wall protein (GRPs)	Structural function in cell wall, scaffold for deposition of cell wall components	Bradi3g47290.1	10214.48	822.79	86.1%
		Bradi3g47307.1	2075.68	363.43	
		Bradi1g26448.1	773.42	201.41	
		Bradi4g13406.1	671.64	329.86	
		Bradi1g78250.1	537.28	193.39	
		Bradi1g54663.1	533.74	103.72	
		Bradi1g12590.1	450.62	78.01	
		Bradi1g28880.1	439.21	79.96	
		Bradi1g64350.2	176.29	20.71	
		Bradi1g28880.1	126.64	114.11	
		Bradi1g26448.1	81.83	19.07	
		Bradi1g09507.1	67.22	8.39	
		Bradi1g09507.1	55.52	21.66	
		Bradi1g64350.2	43.82	5.06	
		Bradi1g73960.1	26.36	6.25	
		Bradi4g21180.1	26.36	4.70	
		Bradi3g27807.1	24.19	3.45	
		Bradi4g30690.1	23.92	2.99	
		Bradi1g73960.1	19.67	4.78	
		Bradi1g57510.2	10.94	0.98	
		Bradi2g05151.2	4.095	2.22	
		Bradi5g17336.1	3.40	1.14	
		Bradi4g21180.1	2.06	1.09	

		Bradi1g05020.1	2.01	1.01	
		Bradi3g09600.1	1.58	0.59	
		Bradi2g58310.1	1.44	0.52	
		Bradi1g56080.2	0.89	0.21	
		Bradi1g34640.1	0.83	0.20	
		Bradi2g28150.2	0.5	0.09	
Expansins	Loosening of plant cell walls	Bradi3g33160.1	803.76	91.19	86.1%
		Bradi2g53580.1	325.39	74.94	
		Bradi2g22290.1	236.48	32.77	
		Bradi3g33106.1	199.76	46.42	
		Bradi5g04120.1	122.65	43.44	
		Bradi1g78350.1	136.53	22.19	
		Bradi3g50740.1	133.07	42.35	
		Bradi3g50750.1	103.57	19.47	
		Bradi1g61190.1	92.98	7.85	
		Bradi1g03640.1	52.28	19.34	
		Bradi3g33150.1	37.34	15.95	
		Bradi5g19340.3	36.52	1.05	
		Bradi3g59460.1	26.29	4.60	
		Bradi1g28130.1	24.38	0.36	
		Bradi3g49850.1	21.76	4.26	
		Bradi3g32297.2	15.36	2.019	
		Bradi1g76260.1	10.48	1.57	
		Bradi1g13787.5	5.81	1.47	
		Bradi1g74740.1	4.04	0.81	
		Bradi1g26990.2	3.63	2.40	
		Bradi1g35830.1	2.98	0.30	
		Bradi5g16497.1	2.72	2.107	
		Bradi1g30050.1	1.23	0.65	
		Bradi1g76270.1	0.94	0.22	
		Bradi2g10320.1	0.58	0.23	

Supp Data file (Data file S1)

RNA-seq data summary file:

Sheet 1: List of 100 highest expressed transcripts identified in *B.~~rachypodium~~ distachyon* coleoptile RNA-seq analysis (based on mean FPKM values from 3 replicates). Members of the XET/XEH family (orange), BURP-domain family (green), AGP family (purple), Glycine-rich cell wall protein family (blue) and Expansin family (red) are highlighted.

Sheet 2: List of 79 *BdWAK* transcripts identified in RNA-seq analysis of *B.~~rachypodium~~ distachyon* coleoptile, as well as those *BdWAKs* not detected. FPKM measurements (based on mean values from 3 replicates), gene accession numbers, along with the chromosomal location of each *BdWAK* are listed. Chromosomal locations within the chromosome 2

BdWAK clusters are highlighted in orange (Bd2:1635336-1700774) and green (Bd2:47168187-47198608).
